# First insights into female sperm storage duration in tardigrades

**DOI:** 10.1002/ece3.9010

**Published:** 2022-06-16

**Authors:** Matteo Vecchi, Justine Chartrain, Simo Puro, Riikka Tynkkynen, Tommi Vuori, Łukasz Michalczyk, Sara Calhim

**Affiliations:** ^1^ Department of Biological and Environmental Science University of Jyväskylä Jyväskylä Finland; ^2^ Department of Invertebrate Evolution, Institute of Zoology and Biomedical Research, Faculty of Biology Jagiellonian University Kraków Poland

**Keywords:** evolution, reproduction, spermatozoa, Tardigrada

## Abstract

Female sperm storage is ubiquitous in the animal kingdom and it has been shown to be linked to several evolutionary processes, from postcopulatory sexual selection to dispersal. Here we report, for the first time, long‐term sperm storage in females of the tardigrade *Macrobiotus polonicus*. Females, isolated after a short contact with a male, were able to use the stored sperm for up to 5 weeks (mean of 2 weeks), which translates to a considerable proportion of female post‐mating longevity under controlled laboratory conditions (60% on average). Our study provides the first insights into the duration of sperm storage, an underexplored feature of the reproductive biology of tardigrades. Additionally, we discuss important considerations for reproductive studies on these non‐model animals.

## Graphical abstract

Tardigrades are an underexplored study system in reproductive and evolutionary biology. We show, for the first time, that female *Macrobiotus polonicus* can use stored sperm from a single mating event for a considerable proportion of their post‐mating lifetime.

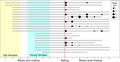

## 1 INTRODUCTION

Sperm storage is a widespread phenomenon in the animal kingdom that can be defined as “the maintenance of sperm inside a female's reproductive tract for an extended period of time” (Orr & Zuk, 2012). Across taxa, there is an incredible variation with respect to storage structure morphology (e.g., crypts, tubules, seminal receptacles, and spermathecae) and to the duration of this storage (from half a day to a decade; Orr & Brennan, 2015). Sperm storage has been linked to several selective benefits (reviewed in Firman et al., 2017; Holt & Fazeli, 2016; Orr & Brennan, 2015; Orr & Zuk, 2012; Pitnick et al., 2009). For example, in female‐biased populations due to sex‐biased mortality or dispersal, female sperm storage could promote population establishment and/or persistence due to increased genetic diversity in the next generations (e.g., Jiménez‐Franco et al., 2020; López‐Sepulcre et al., 2013; Roth & Reinhardt, 2003). There are, however, strong taxonomic biases in the study of evolutionary and ecological consequences of female sperm storage, as most research focuses on fish, birds, and insects (Orr & Brennan, 2015; Pitnick et al., 2020).

Tardigrades are a phylum of microinvertebrates closely related to arthropods (Jørgensen et al., 2018). Our understanding of tardigrade reproductive evolution is still in its infancy, but there is considerable descriptive biology knowledge (reviewed in Altiero et al., 2018; Bertolani & Rebecchi, 1999; Sugiura & Matsumoto, 2021c). Females are iteroparous and oviposition is associated with molting in species that lay smooth eggs into the shed exhuvia, while it is independent of ecdysis in taxa that lay ornamented eggs freely to the environment (Altiero et al., 2018; Bertolani & Rebecchi, 1999; Sugiura & Matsumoto, 2021c). Most taxa are parthenogenetic or gonochoristic/dioecious, with very few recorded hermaphroditic species (Altiero et al., 2018; Bertolani & Rebecchi, 1999). There are currently detailed descriptions of sexual interaction for four gonochoristic species, representing two eutardigrade families (reviewed in Sugiura & Matsumoto, 2021c). These studies all point to a key role of semiochemicals in male and/or ejaculate attraction, and of female behavior in ejaculate intake (Bartel & Hohberg, 2020; Bingemer et al., 2016; Sugiura et al., 2019; Sugiura & Matsumoto, 2021a, 2021b).

There are two major types of sperm storage structures that differ in appearance and histological origin (Bertolani & Rebecchi, 1999): cuticle‐derived external pouches (“seminal receptacles”) in which sperm is stored between mating and oviposition in marine taxa (Jørgensen et al., 1999; Kristensen, 1984), and internal gonadal duct‐derived vesicles (“spermatheca”) that are present in a small subset of limnoterrestrial species (Bertolani, 2001). The former type is reformed at each molting event so females must remate; however, it remains undetermined if females with the latter sperm storage type can use stored sperm to fertilize multiple egg clutches and/or across molting events.

In addition, there is evidence that post‐ejaculation modifications of spermatozoa (reviewed in Pitnick et al., 2020) also occur in tardigrades (Rebecchi, 1997; Sugiura et al., 2019; Sugiura & Matsumoto, 2021a, 2021b; Suzuki & Kristensen, 2014). In Macrobiotidae, the tail is lost soon after the sperm enters the female body (Sugiura et al., 2019; Sugiura & Matsumoto, 2021a, 2021b), and, in species with a clear spermatheca, the sperm stored therein are straightened and packed in bundles (Rebecchi, 1997; cf. Sugiura et al., 2019). In contrast, despite also being straightened inside the seminal receptacle, there is no evidence of a tail reduction in a marine hermaphroditic tardigrade (Suzuki & Kristensen, 2014).

Somewhat surprisingly, it remains unknown how long the sperm remain viable after entering the female reproductive tract in tardigrades. In this study, we aimed to quantify the maximum duration of (and intraspecific variation in) sperm storage ability in a limnoterrestrial tardigrade. Using *Macrobiotus polonicu*s, a species with a clear spermatheca (Poprawa et al., 2015), we tracked the oviposition behavior of isolated females after they were given a short opportunity to mate with a single male.

## 2 MATERIALS AND METHODS

We used a laboratory culture of the moss dwelling parachelan eutardigrade *M. polonicu*s Pilato et al. (2003) (strain AT.002, Figure 2g). The strain originated from individuals extracted from a moss collected in Austria (see Stec, Vecchi, Calhim, et al., 2021 for details), and has been kept in 5 cm‐diameter plastic Petri dishes with a scraped bottom (to promote motility) filled with mineral water and fed *ad libitum* with algae (*Chlorococcum hpnosporum* and *Chlorella* sp.; Sciento UK) and rotifers (*Lecane* sp.; provided by Dr Edyta Fiałkowska, Jagiellonian University, Poland) since 2014. The cultures were kept in a climate‐controlled chamber at 16°C, in a 22 + 2 h D:L regime. Half of the medium was partially changed weekly. Because of their transparent cuticle, the sex and reproductive maturity can be determined non‐invasively using light microscopy (LM): mature males can be recognized thanks to the presence of motile sperm in the testis; mature females can be identified by the presence of large oocytes with a significant amount of yolk in the ovary (maturation stage 3 or 4; Rebecchi & Bertolani, 1994). To visualize tardigrade gonads in vivo, individuals were placed in a 5 μl drop of spring water in a channel created by sticking two parallel strips of adhesive plastic tape onto a microscope slide. Those tape strips prevent the individual from being crushed when a coverslip is placed for observation under LM at 400× magnification.

### 2.1 Mating experiment and oviposition monitoring

Only virgin females were used in the experiments because in a pilot assay almost all (*n* = 34/35) randomly selected females from the main stock had sperm in their spermathecae (spermatozoa identified using Orcein staining; Bertolani, 1971; Figure 2a–f), despite having only stage 1 ovary development (i.e., ovary containing undifferentiated cells very similar to each other; Rebecchi & Bertolani, 1994). Thus, virgin females were obtained by isolating eggs or hatchlings (sexually immature first instar) in 3 cm‐diameter plastic dishes, kept in the same conditions as the main culture. The males used in this experiment were extracted from the main culture (hence unlikely to be virgins) and kept in male‐only groups for a minimum of 7 days (in the same conditions as the main culture). Males were identified non‐invasively by the examination of the gonad under LM (see above). Before the mating trial, sexual maturity was confirmed for both sexes (see above). None of the isolated females laid eggs before the mating trials. The mating trials were conducted by placing each one of the *n* = 22 sexually mature virgin females (*n* = 9 aged 3–4 weeks and *n* = 13 aged 7–8 weeks) together with a single randomly selected sexually mature male in 5 mm‐diameter wells (of a 96 well plate) with a 1% agar base and spring water for 1.5 h in the dark. Each isolated male was used only once. After the mating trial, females were placed back in individual 3 cm‐diameter dishes to be monitored in isolation. Mating and oviposition were conducted at 21(±1)°C. Females were checked regularly for the presence of laid eggs: the first check was conducted 24 h post‐mating, the second check 1 week post‐mating, and all subsequent ones at weekly intervals until the female was dead. The eggs were counted and removed at each check.

### 2.2 Statistical analyses

All statistical analyses were conducted in R v.4.1.0 (R Core Team, 2021). Due to the small sample size for this observational study, we opted for Bayesian approaches. We ran generalized linear models using the “brm” function from the “brms” package v.2.15.0 (Bürkner, 2017). Due to the bimodality in the age of the females used, this predictor is included as a two‐level factor: young (3–4 weeks; *n* = 9) and old (7–8 weeks; *n* = 13). We used default priors for each response distribution type: Bernoulli (logit link) distribution for the oviposition occurrence (1 = did and 0 = did not lay eggs); Negative binomial (log link) distribution for a number of laid eggs; geometric (log link) for longevity and oviposition interval. Uncertainty in the date of events was incorporated in the models with the function “cens.” Oviposition rate was estimated using the number of days between checks as an offset term in the model. The results are presented as back‐transformed Bayesian posterior means and 95% high‐density interval (HDI) using the “mean_hdi” function from the “tidybayes” package v. 3.0.0 (Kay, 2021), computed from *n* = 3 well converging and not autocorrelated chains with *n* = 2000 saved iterations each. Bayesian *p*‐values were calculated according to Makowski et al. (2019) with the R package “bayestestR v. 0.3.0” (Makowski et al., 2019).

## 3 RESULTS AND DISCUSSION

Our study provides the first account of how long sperm are stored in a tardigrade that has a clear sperm storage structure. In addition, it offers an initial look at how much intraspecific variation there is in this trait. Despite the challenges of working with non‐model organisms, our results unlock several avenues for further research into the role of sperm storage on evolution and behavior in this animal group. Therefore, we discuss the theoretical implications of the currently limited information (from this study and literature) together with key methodological considerations for future research.

### 3.1 Oviposition behavior as a proxy for sperm transfer success in tardigrades

**FIGURE 1:**
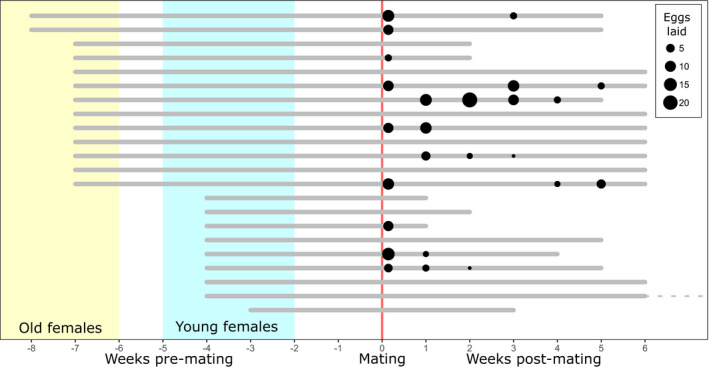
Life histories of females which were kept in isolation after a short mating opportunity as virgins. The start and end of the gray bars indicate the hatching and death of single individuals (the dotted gray line indicates the one female who lived beyond the duration of the experiment)

There was a considerable variation in oviposition behavior and fecundity across females (Figure 1). Specifically, only half of the females (11/22) laid eggs and total fecundity ranged between 3 and 46 eggs (median = 15 eggs). Eggs were laid in 1–4 separate (but not always sequential) weekly “batches” of a median of 6 eggs each (range: 1–22 eggs). On average, females that laid at least two batches did so at an average interval of about 9 days (*n* = 8 females, Bayesian mean estimate [95% HDI] = 9.2 [3.1, 16.4] days). Interestingly, female age at mating was not significantly associated either with the probability of laying (mean [95% HDI]: young females (≤4 weeks‐old) = 0.56 [0.26, 0.85]; old females (≥7 weeks‐old) = 0.46 [0.20, 0.71]); Bayesian *p*‐value = .629), nor with total fecundity (mean [95% HDI]: young = 21.4 [8.81, 35.5] eggs; old = 13.8 [7.83, 27.4] eggs; Bayesian *p*‐value = .357). Lastly, most laying females (82%, *n* = 9/11) laid their first batch of eggs in the first 24 h, with the remaining ones (*n* = 2/11) laying their first eggs within the first week. Females that did not lay eggs in the first week, did not oviposit later on either.

Gonochoristic eutardigrade females without access to males have been shown to reabsorb rather than lay unfertilized eggs (Baumann, 1970; Bingemer et al., 2016). Monitoring oviposition behavior in *M. polonicus* provides two useful methodological time thresholds for future research. First, a 1‐week period without oviposition for a female with mature oocytes after contact with a sexually mature male can be used as a proxy for the lack of insemination success in this species. A similar time threshold can be expected in other closely related macrobiotids with known or suspected presence of spermatheca (i.e. taxa clustered in subclade B *in* Stec, Vecchi, Calhim, et al., 2021, Stec, Vecchi, Dudziak, et al., 2021). Monitoring oviposition after the experimentally controlled mating encounters beyond the first batch of eggs (see what has been previously done e.g., Sugiura et al., 2019; Sugiura & Matsumoto, 2021a) would provide key data for interspecific variation in sperm storage ability in Macrobiotidae. Second, despite our limited sample size, we found that subsequent ovipositions using stored sperm occurred, on average, at 1.5‐week intervals. Therefore, a 2‐week period in isolation seems a conservative threshold to ensure that sexually mature females extracted from stock cultures do not store (viable) sperm. At least for our study species, it is time‐consuming to rear sexually mature virgin individuals: it takes 6 weeks for hatchlings reared in isolation to reach sexual maturity, and only one‐third reach that point (J. Chartrain, *pers. obs*.). Therefore, this period could be a useful alternative source of “sperm‐free” females for experimental research.

### 3.2 A possible role for pre‐mating sexual selection in tardigrades

The mating success of randomly assigned sexually mature *M. polonicus* pairs is considerably lower than would be expected based on the behavioral observation studies in three other macrobiotids (*Macrobiotus shonaicus* Stec et al., 2018, *Paramacrobiotus metropolitanus* Sugiura et al., 2022, and *Mesobiotus* sp.; reviewed in Sugiura & Matsumoto, 2021c). In the latter work, the complete mating interaction was initiated soon after the two individuals were placed close together, and completed within 30 min (Sugiura et al., 2019; Sugiura & Matsumoto, 2021a). Thus, these studies suggest that these tardigrades are eager to mate under laboratory conditions. In our study, in contrast, only half the females laid eggs, despite a less disturbed (i.e., unobserved), and three times longer, access to a sexually mature partner. Perhaps two methodological distinctions can explain this discrepancy: in our study, all sexually mature females were virgins, and it is the only taxa with a clear sperm storage structure (*see* Sugiura et al., 2019). Although tardigrade males have a key role in mate searching and initiating sexual interactions, females seem to have a strong influence on sperm transfer success through an active body movement for sperm intake (Bingemer et al., 2016; Sugiura et al., 2019; Sugiura & Matsumoto, 2021a). Therefore, our results suggest that despite having a mature gonad, virgin macrobiotid females show variation in readiness/willingness/ability to mate/use sperm of the first male they encounter. Note that the fact that a great majority of females in our *M. polonicus* main stocks have their spermathecae filled with sperm (97%, *n* = 34/35), is evidence that females can readily secure fertilization when the choice is abundant. This phenomenon warrants further investigation, as it suggests pre‐mating sexual selection could occur in this animal group.

### 3.3 Spermatheca sperm organization—do multiple bundles reflect polyandry?

**FIGURE 2:**
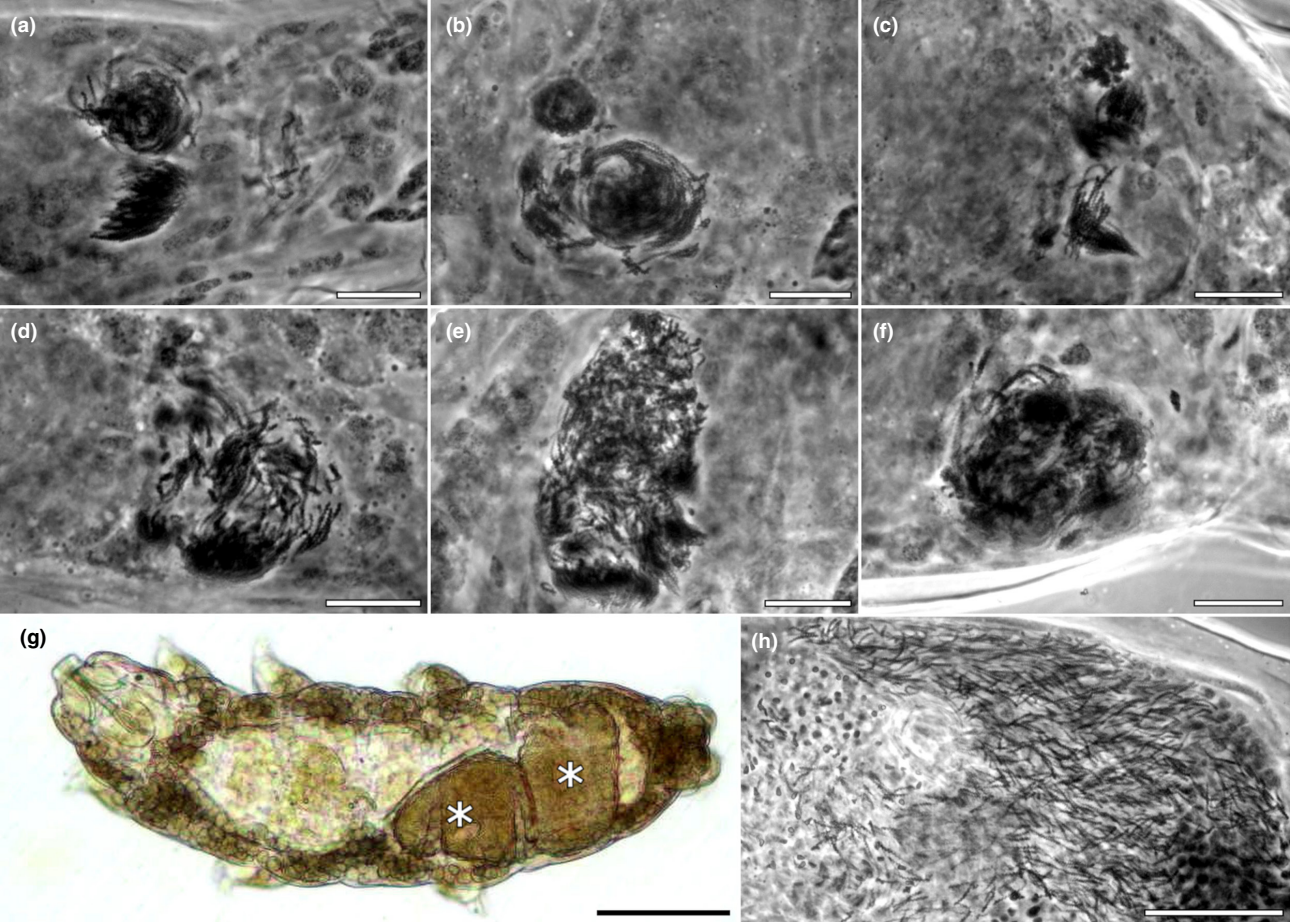
Sperm arrangement inside females and a male. (a–f) Sperm bundles inside female spermathecae (Orcein stain in light microscopy, LM), scale bar 10 μm. (g) In toto female at ovary maturation stage 4 (LM) showing mature oocytes (asterisks), scale bar 100 μm. (h) Sperm inside a male gonad (Orcein stain in LM), scale bar 20 μm

We found considerable intraspecific variation in how sperm cells are organized inside the spermatheca (Figure 2a–f). Compared to the loose arrangement of sperm inside the male gonad (Figure 2h), sperm inside the females were organized in relatively tightly packed single (Figure 2e–f) or multiple (Figure 2a–d) bundles. The presence of multiple sperm bundles (e.g., Rebecchi, 1997; Figure 2a–c) is intriguing and warrants further experimental research. Evolutionary speaking, it could be the result of multiple mating events (with the same or different males) or multiple ejaculate uptakes from the same mating interaction (e.g., Sugiura & Matsumoto, 2021a). At a mechanistic level, the variation in the degree of sperm organization inside the female spermatheca (Figure 2a–f) could be due to three, non‐mutually exclusive, processes: weak control mechanisms for sperm placement; variation in the time since ejaculate uptake (across females and across bundles within female); or a methodological artifact because of varying spatial orientation of the animals on the microscope slide, or of physical distortion from the coverslip pressure. Unfortunately, Orcein staining is a simple but invasive imaging technique, which kills the animals and thus limits experimental research options. Therefore, the development of live (fluorescent) staining techniques such as those used in other taxa (e.g. Manier et al., 2010; Marie‐Orleach et al., 2014) and/or paternity assessment tools could revolutionize the study of post‐copulatory mechanisms in tardigrades.

### 3.4 Sperm storage index

The last observed oviposition event by single mated isolated females varied considerably (Figure 1), with the observed maximum of 5 weeks post‐mating (mean [95% HDI] = 15.7 [7.07, 25.5] days after mating). Moreover, after a single bout of mating as virgins, isolated females stored sperm for a substantial proportion of their active reproductive lifetime (mean estimate [95% HDI] = 57.0 [51.7, 62.4]%). Importantly, accounting for female age at mating, there was no difference in longevity between females that did and did not lay eggs (mean [95% HDI]: laid = 73.0 [34.3, 120] days; not laid = 80.5 [37.5, 137] days; Bayesian *p*‐value = .841).

To place tardigrade sperm storage ability within a wider perspective across animals, we followed the guidelines developed by Orr and Brennan (2015). In their paper, Orr & Brennan suggested the use of a sperm storage index (SSI) as a way to obtain a standardized method to evaluate sperm storage occurrence across taxa irrespective of the amount of the available data. The SSI is based on 12 criteria, and each is given a value depending on the strength of evidence (Orr & Brennan, 2015). Table 1 provides a summary of what is known for each criterion for tardigrades in general and/or for our study species and related taxa, by combining published literature and the results from our study. Importantly, we also use the SSI criteria framework to highlight knowledge gaps and offer suggestions to tackle them. It is clear that many avenues are yet to be explored, especially those linked to molecular and physiological aspects of sperm‐female interactions. Since female tardigrades play a key behavioral role during sperm transfer/insemination (reviewed in Sugiura & Matsumoto, 2021c), and a substantial modification of sperm morphology takes place soon after insemination in macrobiotids (i.e., greatly reduced flagellum and thus motility; Rebecchi, 1997; Sugiura & Matsumoto, 2021c), it would not be surprising if there are also other physiological adaptations to long‐term storage in tardigrades, such as sperm nourishment (see Table 1; Orr & Brennan, 2015).

**TABLE 1:** Current knowledge of sperm storage in tardigrades based on the 12 criteria developed by Orr and Brennan (2015), with suggested future research possibilities

Criteria	Score	Comments/future work
Sperm live longer in the female than in similar conditions not within the female	0	Currently, there are no data on the physiological features of (macrobiotid) tardigrade female reproductive tract. Therefore, we cannot recreate “similar conditions” artificially. The same lack of knowledge applies to the non‐sperm component of the tardigrade ejaculate.
Sperm viable after storage	1	After a single mating opportunity, females without further access to males, continue to lay eggs cf. reabsorb them (this study). The next step would be to investigate the hatching success across subsequent clutches to determine if there is a loss of sperm viability with time.
Storage structures (organs)	1	This study species (Figure 2a–f and Poprawa et al., 2015) and other members of the subclade B of the genus *Macrobiotus* (Rebecchi, 1997; Stec, Vecchi, Calhim, et al., 2021; Stec, Vecchi, Dudziak, et al., 2021) have a spermatheca.
Sperm stored in particular regions	1	The spermatheca is in a specific region of the female body, with the opening located between the cloaca and the hindgut (Poprawa et al., 2015). It would be important to assess how long sperm can be used in macrobiotid taxa outside subclade B (see Stec, Vecchi, Calhim, et al., 2021 and Stec, Vecchi, Dudziak, et al., 2021 for a genus‐wide phylogenetic perspective).
Multiple (types) of sperm storage are evident	NA	It is unclear at what taxonomic level this criterion should be applied. Across the phylum, there are two types of sperm storage structures (external and internal) but each is restricted to a given Class (e.g., reviewed in Bertolani & Rebecchi, 1999).
Organized arrangement of sperm (e.g., heads aligned)	0.5	Sperm in the spermatheca of *Macrobiotus* sp. are organized (this study Figure 2a–f; Poprawa et al., 2015; Rebecchi, 1997).
Female immune suppression (localized)	0	There are no studies on the potential immune response suppression in female tardigrades.
Sperm receive nourishment	0	The relatively long time period in storage in *Macrobotus polonicus* (max = 5 weeks; this study) suggests that some form of nourishment could be possible. However, this has not yet been studied.
Specialized sperm (ejaculate) biochemistry	0	As with the female reproductive tract, there are currently no data on tardigrade ejaculate biochemistry.
Ovulation is only at the end of the sperm storage period	1	Ovulation is not restricted to the end of the storage period (this study).
Sperm activity diminished during storage but returns at termination of storage (i.e. synchronization with female reproductive pattern)	0.5	Spermatozoa undergo almost complete tail loss upon entering the female reproductive tract (reviewed in Sugiura & Matsumoto, 2021c) and are immotile while in storage (Rebecchi, 1997). However, fertilization could require sperm activity even if this does not include the recovery of flagellar motility.
Sperm moved in a targeted manner to the storage site	0	There is some evidence of targeted sperm movement toward the female cloaca opening after ejaculation (reviewed in Sugiura & Matsumoto, 2021c) but there are no data with respect to targeted movement inside the female reproductive tract. Development of live staining techniques, especially sperm‐specific, are needed to assess this criterion.

*Note*: Scores for each criterion are based on the degree of evidence (0 = none, 0.5 = some, 1 = conclusive). A given taxon's sperm storage index (SSI) is calculated by multiplying the sum of scores by the number of criteria that could be assessed, and then dividing the result by the total number of criteria available (Orr & Brennan, 2015). Consequently, the SSI for *Macrobiotus polonicus* = (5*6/12) = 2.5, with half of the criteria requiring further study.

We found considerable intraspecific variability in how (Figure 2a–f) and how long (Figure 1) sperm are stored in *M. polonicus*. It is therefore likely that across species these differences also occur. Orcein staining provides a snapshot of where and how spermatozoa are found inside the female reproductive tract. Although several studies have used this technique, the interpretation of the images seems inconsistent, perhaps due to any of many factors that can affect the latter, such as the time interval since mating, specimen quality, and sample size. For example, *M. shonaicus*, *P. metropolitanus*, and *Mesobiotus* sp. are said to have a spermatheca (Sugiura et al., 2019; Sugiura & Matsumoto, 2021a). However, only a very few (*n* = 3 and 5) females were imaged and only shortly (5 min) after mating (Sugiura et al., 2019; Sugiura & Matsumoto, 2021a). Those images show sperm spanning relatively long stretches of the reproductive tract, being particularly disorganized in *P. metropolitanus*. In these studies, females laid the first batch of eggs within 1–3 days, but no oviposition data beyond that were provided. Therefore, published observations for these three macrobiotids are insufficient to rule out that sperm is present inside the female for only a relatively short period between mating and the first/only oviposition event.

### 3.5 Sperm storage nomenclature

We propose the term “spermatheca” *sensu stricto* to reflect the functional ability to store sperm across multiple oocyte maturation cycles. As shown here for *M. polonicus*, evidence for the presence of a spermatheca can be assessed by monitoring oviposition cycles of females kept isolated from males (after an expected mating event) for several weeks. Although controlled matings using virgin females would be preferred (as it was done in this study), using stock population females is a feasible alternative. Furthermore, the presence of sperm in the spermatheca of stock population individuals with an immature ovary (e.g., using Orcein staining) is suggestive, albeit not conclusive, evidence that sperm could have been retained from the previous oviposition cycle, and/or that they might be stored for the full duration of the current oocyte development. A recent phylogenetic analyses of the genus *Macrobiotus* places most species with indications of the presence of spermatheca in a subclade B sensu Stec, Vecchi, Calhim, et al. (2021) and Stec, Vecchi, Dudziak, et al. (2021). None of the three macrobiotid taxa with detailed descriptions of sexual interactions (Sugiura & Matsumoto, 2021c) belong to this subclade. Thus, it would be very useful to have a more detailed anatomical resolution of the key area of the female reproductive tract where spermatheca might be present, coupled with the monitoring of lifetime oviposition patterns of post‐mating‐isolated females across Macrobiotidae. Perhaps there is some degree of anatomical (and/or physiological) difference between reproductive tracts of tardigrade taxa that hold sperm only short‐ *versus* long‐term, which is at the most extreme illustrated by the two types of sperm storage organs in *Drosophila* fruitflies (Pitnick et al., 1999).

### 3.6 Ecological consequences of sperm storage in tardigrades

Most limnoterrestrial tardigrades are known for the ability to survive desiccation through anhydrobiosis (for review see Wełnicz et al., 2011 and Rebecchi et al., 2020), which is thought to aid dispersal by wind or larger animals. We know that long‐term anhydrobiosis affects the recovery time for fully functional sperm in male macrobiotid tardigrades (Vecchi et al., in prep), thus it would be interesting to test if sperm stored in females may survive desiccation too. If this is indeed the case, spermathecae may play a significant role in tardigrade evolution and dispersal. Specifically, a single mated female with stored sperm in her spermatheca, similarly to a parthenogenetic female (but not a gonochoristic one without a long‐term storage organ), could be able to establish a population after colonizing a new moss cushion without further matings (e.g., up to 46 eggs have been produced by a single mating in *M. polonicus*; Figure 1). Furthermore, if males and females differ in anhydrobiotic survival, the benefits of storing sperm maybe even greater. In the predatory bug (*Nabis rugosus* Linnaeus, 1758), hibernating females with low resources bias investment into the maintenance of stored sperm rather than in egg production since males have lower hibernation survival (Roth & Reinhardt, 2003). Perhaps a similar pattern occurs in anhydrobiosis‐competent and sperm‐storing tardigrades. Any potential fitness benefits of sperm storage (such as securing fertilization over long periods of time or dispersal advantage) may explain the evolution of spermathecae despite potential fitness costs, which may include energetic expenses needed to maintain the spermatozoa. Last but not least, our current knowledge of the molecular mechanisms behind anhydrobiosis resistance in tardigrades (e.g., reviewed in Hibshman et al., 2020; Rebecchi et al., 2020; Schill & Hengherr, 2018; Wełnicz et al., 2011) could inspire a new line of “Omics” studies in the context of sperm storage. For example, the heat shock protein HSp70 is upregulated not only during rehydration and recovery phases in four anhydrobiosis‐competent tardigrade species (reviewed in Hibshman et al., 2020), but also in the sperm storage tissue epithelium of birds as part of complex sperm–female molecular interactions (Long et al., 2003). The application of RNA interference techniques, recently used in the study of stress response of tardigrades (e.g., Giovannini et al., 2022), could prove to be key to the investigation of molecular interactions between gametes and reproductive tissues.

## 4 CONCLUDING REMARKS

This is the first, albeit preliminary, study on sperm storage duration in the phylum Tardigrada. Unsurprisingly, many open questions remain (see Table 1), such as how widespread long‐term sperm storage is among limnoterrestrial tardigrades, and what consequences it has for ecological (e.g., dispersal and colonization constraints) and evolutionary (e.g., post‐copulatory sexual selection) processes. Most reproductive evolution research focuses on just a handful of taxa, despite suggestions that there could be “benefits of carrying out descriptive studies of the fascinating reproductive biology of diverse taxa” (Pitnick et al., 2020). We hope that this paper will inspire further evolutionary research in this poorly explored phylum of microscopic animals.

## AUTHOR CONTRIBUTIONS

**Matteo Vecchi:** Conceptualization (lead); data curation (equal); formal analysis (supporting); investigation (equal); methodology (equal); project administration (equal); software (equal); supervision (equal); validation (equal); visualization (lead); writing – original draft (equal); writing – review and editing (lead). **Justine Chartrain:** Investigation (equal); writing – review and editing (supporting). **Simo Puro:** Investigation (equal); writing – review and editing (supporting). **Riikka Tynkkynen:** Investigation (equal); writing – review and editing (supporting). **Tommi Vuori:** Investigation (equal); writing – review and editing (supporting). **Łukasz Michalczyk:** Conceptualization (supporting); resources (equal); visualization (supporting); writing – review and editing (lead). **Sara Calhim:** Conceptualization (lead); data curation (equal); formal analysis (lead); funding acquisition (lead); methodology (equal); project administration (equal); resources (equal); software (equal); supervision (equal); validation (equal); visualization (supporting); writing – original draft (equal); writing – review and editing (lead).

## CONFLICT OF INTEREST

The authors have no competing interests to declare.

## Data Availability

The datasets, annotated R script and complete analyses output for this study will be made available on Dryad as an .html rendered RMarkdown file (DOI: https://doi.org/10.5061/dryad.fn2z34txb).

## References

[ece39010-bib-0001] Altiero, T. , Suzuki, A. C. , & Rebecchi, L. (2018). Reproduction, development and life cycles. In R. O. Schill (Ed.), Water bears: The biology of tardigrades (pp. 211–247). Springer.

[ece39010-bib-0002] Bartel, S. , & Hohberg, K. (2020). Experimental investigations on the partner‐finding behaviour of Isohypsibius dastychi (Isohypsibiidae: Tardigrada). Zoological Journal of the Linnean Society, 188(3), 878–886.

[ece39010-bib-0003] Baumann, H. (1970). Lebenslauf und Lebensweise von *Macrobiotus hufelandii* Schultze (Tardigrada). Veroff Uberseemus Bremen, 4, 29–43.

[ece39010-bib-0004] Bertolani, R. (1971). Contributo alla cariologia dei Tardigradi. Osservazioni su Macrobiotus hufelandii. Atti della Accademia Nazionale dei Lincei. Classe di Scienze Fisiche, Matematiche e Naturali. Rendiconti, 50(6), 772–775.

[ece39010-bib-0005] Bertolani, R. (2001). Evolution of the reproductive mechanisms in tardigrades—A review. Zoologischer Anzeiger–A Journal of Comparative Zoology, 240(3–4), 247–252.

[ece39010-bib-0006] Bertolani, R. , & Rebecchi, L. (1999). Tardigrada. In E. Knobil & J. D. Neill (Eds.), Encyclopedia of reproduction (Vol. 4, pp. 703–717). Academic Press.

[ece39010-bib-0007] Bingemer, J. , Hohberg, K. , & Schill, R. O. (2016). First detailed observations on tardigrade mating behaviour and some aspects of the life history of Isohypsibius dastychi Pilato, Bertolani & Binda 1982 (Tardigrada, Isohypsibiidae). Zoological Journal of the Linnean Society, 178(4), 856–862.

[ece39010-bib-0008] Bürkner, P. C. (2017). Brms: An R package for Bayesian multilevel models using Stan. Journal of Statistical Software, 80(1), 1–28.

[ece39010-bib-0010] Firman, R. C. , Gasparini, C. , Manier, M. K. , & Pizzari, T. (2017). Postmating female control: 20 years of cryptic female choice. Trends in Ecology and Evolution, 32, 368–382. 28318651 10.1016/j.tree.2017.02.010 PMC5511330

[ece39010-bib-0011] Giovannini, I. , Boothby, T. C. , Cesari, M. , Goldstein, B. , Guidetti, R. , & Rebecchi, L. (2022). Production of reactive oxygen species and involvement of bioprotectants during anhydrobiosis in the tardigrade *Paramacrobiotus spatialis* . Scientific Reports, 12, 1938. 35121798 10.1038/s41598-022-05734-6 PMC8816950

[ece39010-bib-0014] Hibshman, J. D. , Clegg, J. S. , & Goldstein, B. (2020). Mechanisms of desiccation tolerance: Themes and variations in brine shrimp, roundworms, and tardigrades. Frontiers in Physiology, 11. 10.3389/fphys.2020.592016 PMC7649794 33192606

[ece39010-bib-0015] Holt, W. V. , & Fazeli, A. (2016). Sperm storage in the female reproductive tract. Annual Review of Animal Biosciences, 4, 291–310. 26526545 10.1146/annurev-animal-021815-111350

[ece39010-bib-0016] Jiménez‐Franco, M. V. , Giménez, A. , Rodríguez‐Caro, R. C. , Sanz‐Aguilar, A. , Botella, F. , Anadón, J. D. , Wiegand, T. , & Graciá, E. (2020). Sperm storage reduces the strength of the mate‐finding Allee effect. Ecology and Evolution, 10(4), 1938–1948. 32128127 10.1002/ece3.6019 PMC7042743

[ece39010-bib-0017] Jørgensen, A. , Kristensen, R. M. , & Møbjerg, N. (2018). Phylogeny and integrative taxonomy of Tardigrada. In R. O. Schill (Ed.), Water bears: The biology of tardigrades (pp. 95–114). Springer.

[ece39010-bib-0018] Jørgensen, A. , Mobjerg, N. , & Kristensen, R. M. (1999). Ultrastructural studies on spermiogenesis and postcopulatory modifications of spermatozoa of Actinarctus doryphorus Schulz, 1935 (Arthrotardigrada: Halechiniscidae). Zoologischer Anzeiger, 238(3–4), 235–257.

[ece39010-bib-0019] Kay, M. (2021). Tidybayes: Tidy data and Geoms for Bayesian models. doi: 10.5281/zenodo.1308151, R package version 3.0.0, http://mjskay.github.io/tidybayes/

[ece39010-bib-0020] Kristensen, R. M. (1984). On the biology of *Wingstrandarctus corallinus* nov. gen. Et spec., with notes on the symbiontic bacteria in the subfamily Florarctinae (Arthrotardigrada). Videnskabelige Meddelelser fra Dansk Naturhistorisk Forening, 145, 201–218.

[ece39010-bib-0021] Long, E. L. , Sonstegard, T. S. , Long, J. A. , Van Tassell, C. P. , & Zuelke, K. A. (2003). Serial analysis of gene expression in Turkey sperm storage tubules in the presence and absence of resident sperm. Biology of Reproduction, 69, 469–474. 12672662 10.1095/biolreprod.102.015172

[ece39010-bib-0051] Linnaeus, C. (1758). Systema naturae (Vol. 1, No. part 1, p. 532). Laurentii Salvii.

[ece39010-bib-0022] López‐Sepulcre, A. , Gordon, S. P. , Paterson, I. G. , Bentzen, P. , & Reznick, D. N. (2013). Beyond lifetime reproductive success: The posthumous reproductive dynamics of male Trinidadian guppies. Proceedings of the Royal Society B: Biological Sciences, 280(1763), 20131116. 10.1098/rspb.2013.1116 PMC3774245 23740786

[ece39010-bib-0023] Makowski, D. , Ben‐Shachar, M. S. , Chen, S. H. , & Lüdecke, D. (2019). Indices of effect existence and significance in the Bayesian framework. Frontiers in Psychology, 10, 2767. 31920819 10.3389/fpsyg.2019.02767 PMC6914840

[ece39010-bib-0024] Makowski, D. , Ben‐Shachar, M. S. , & Lüdecke, D. (2019). bayestestR: Describing effects and their uncertainty, existence and significance within the Bayesian framework. Journal of Open Source Software, 4, 1541.

[ece39010-bib-0025] Manier, M. K. , Belote, J. M. , Berben, K. S. , Novikov, D. , Stuart, W. T. , & Pitnick, S. (2010). Resolving mechanisms of competitive fertilization success in *Drosophila melanogaster* . Science, 328(5976), 354–357. 20299550 10.1126/science.1187096

[ece39010-bib-0026] Marie‐Orleach, L. , Janicke, T. , Vizoso, D. B. , Eichmann, M. , & Schärer, L. (2014). Fluorescent sperm in a transparent worm: Validation of a GFP marker to study sexual selection. BMC Evolutionary Biology, 14, 148. 24980980 10.1186/1471-2148-14-148 PMC4107727

[ece39010-bib-0027] Orr, T. J. , & Brennan, P. L. (2015). Sperm storage: Distinguishing selective processes and evaluating criteria. Trends in Ecology & Evolution, 30(5), 261–272. 25843274 10.1016/j.tree.2015.03.006

[ece39010-bib-0028] Orr, T. J. , & Zuk, M. (2012). Sperm storage. Current Biology, 22(1), R8–R10. 22240479 10.1016/j.cub.2011.11.003

[ece39010-bib-0029] Pilato, G. , Kaczmarek, L. , Michalczyk, L. , & Lisi, O. (2003). Macrobiotus polonicus, a new species of Tardigrada from Poland (Eutardigrada: Macrobiotidae,‘hufelandi group’). Zootaxa, 258, 1–8.

[ece39010-bib-0030] Pitnick, S. , Markow, T. , & Spicer, G. S. (1999). Evolution of multiple kids of female sperm‐storage organs in *drosophila* . Evolution, 53, 18041822. 10.1111/j.1558-5646.1999.tb04564.x 28565462

[ece39010-bib-0031] Pitnick, S. , Wolfner, M. F. , & Dorus, S. (2020). Post‐ejaculatory modifications to sperm (PEMS). Biological Reviews, 95(2), 365–392. 31737992 10.1111/brv.12569 PMC7643048

[ece39010-bib-0032] Pitnick, S. , Wolfner, M. F. & Suarez, S. S. (2009). Ejaculate‐female and sperm‐female interactions. In Sperm biology. Birkhead TR , Hosken D , & Pitnicks S (eds). Elsevier (pp. 247–304).

[ece39010-bib-0033] Poprawa, I. , Schlechte‐Wełnicz, W. , & Hyra, M. (2015). Ovary organization and oogenesis in the tardigrade Macrobiotus polonicus Pilato, Kaczmarek, Michalczyk & Lisi, 2003 (Eutardigrada, Macrobiotidae): Ultrastructural and histochemical analysis. Protoplasma, 252(3), 857–865. 25380721 10.1007/s00709-014-0725-x

[ece39010-bib-0049] R Core Team . (2021). R: A language and environment for statistical computing. R Foundation for Statistical Computing. https://www.R‐project.org/

[ece39010-bib-0034] Rebecchi, L. (1997). Ultrastructural study of spermiogenesis and the testicular and spermathecal spermatozoon of the gonochoristic tardigrade *Xerobiotus pseudohufelandi* (Eutardigrada, Macrobiotidae). Journal of Morphology, 234(1), 11–24. 29852653 10.1002/(SICI)1097-4687(199710)234:1<11::AID-JMOR2>3.0.CO;2-Q

[ece39010-bib-0035] Rebecchi, L. , & Bertolani, R. (1994). Maturative pattern of ovary and testis in eutardigrades of freshwater and terrestrial habitats. Invertebrate Reproduction & Development, 26(2), 107–117.

[ece39010-bib-0036] Rebecchi, L. , Boschetti, C. , & Nelson, D. R. (2020). Extreme‐tolerance mechanisms in meiofaunal organisms: A case study with tardigrades, rotifers and nematodes. Hydrobiologia, 847(12), 2779–2799.

[ece39010-bib-0037] Roth, S. , & Reinhardt, K. (2003). Facultative sperm storage in response to nutritional status in a female insect. Proceedings of the Royal Society B: Biological Sciences, 270, S1–S4. 10.1098/rsbl.2003.0008 PMC1698029 12952635

[ece39010-bib-0038] Schill, R. O. , & Hengherr, S. (2018). Environmental adaptations: desiccation tolerance. In Water bears: The biology of tardigrades (pp. 273–293). Springer.

[ece39010-bib-0039] Stec, D. , Arakawa, K. , & Michalczyk, Ł. (2018). An integrative description of Macrobiotus shonaicus sp. nov. (Tardigrada: Macrobiotidae) from Japan with notes on its phylogenetic position within the hufelandi group. PLoS One, 13(2), e0192210. 10.1371/journal.pone.0192210 29489835 PMC5830310

[ece39010-bib-0040] Stec, D. , Vecchi, M. , Calhim, S. , & Michalczyk, Ł. (2021). New multilocus phylogeny reorganises the family Macrobiotidae (Eutardigrada) and unveils complex morphological evolution of the *Macrobiotus hufelandi* group. Molecular Phylogenetics and Evolution, 160, 106987. 33059070 10.1016/j.ympev.2020.106987

[ece39010-bib-0041] Stec, D. , Vecchi, M. , Dudziak, M. , Bartels, P. J. , Calhim, S. , & Michalczyk, Ł. (2021). Integrative taxonomy resolves species identities within the *Macrobiotus pallarii* complex (Eutardigrada: Macrobiotidae). Zoological Letters, 7(1), 1–45. 34044886 10.1186/s40851-021-00176-w PMC8162020

[ece39010-bib-0042] Sugiura, K. , & Matsumoto, M. (2021a). Reproduction of *Mesobiotus*: Comparison of morphology and behavior in the family Macrobiotidae (Tardigrada: Eutardigrada). Zoological Science, 38(5), 444–450. 34664919 10.2108/zs210045

[ece39010-bib-0043] Sugiura, K. , & Matsumoto, M. (2021b). Spermatozoa morphology changes during reproduction and first observation of acrosomal contact in two dioecious species of Macrobiotidae (Tardigrada: Eutardigrada). Zygote, 29(1), 42–48. 32914733 10.1017/S0967199420000490

[ece39010-bib-0044] Sugiura, K. , & Matsumoto, M. (2021c). Sexual reproductive behaviours of tardigrades: A review. Invertebrate Reproduction & Development, 65(4), 1–9.

[ece39010-bib-0045] Sugiura, K. , Matsumoto, M. , & Kunieda, T. (2022). Description of a model tardigrade Paramacrobiotus metropolitanus sp. nov. (Eutardigrada) from Japan with a summary of its life history, reproduction and genomics. Zootaxa, 5134(1), 92–112. 10.11646/zootaxa.5134.1.4 36101075

[ece39010-bib-0046] Sugiura, K. , Minato, H. , Suzuki, A. C. , Arakawa, K. , Kunieda, T. , & Matsumoto, M. (2019). Comparison of sexual reproductive behaviors in two species of Macrobiotidae (Tardigrada: Eutardigrada). Zoological Science, 36(2), 120–127. 31120646 10.2108/zs180103

[ece39010-bib-0047] Suzuki, A. C. , & Kristensen, R. M. (2014). Spermatozoa in the reproductive system of a hermaphroditic marine tardigrade, *Orzeliscus belopus* (Tardigrada: Arthrotardigrada). Zoologischer Anzeiger–A Journal of Comparative Zoology, 253(6), 497–511.

[ece39010-bib-0050] Vecchi, M. , Vuori, T. , Monttinen, M. , Bruneaux, M. , Michalczyk, Ł. , & Calhim, S. Effect of anhydrobiosis on tardigrade male gametes. In prep.

[ece39010-bib-0048] Wełnicz, W. , Grohme, M. A. , Kaczmarek, Ł. , Schill, R. O. , & Frohme, M. (2011). Anhydrobiosis in tardigrades—The last decade. Journal of Insect Physiology, 57(5), 577–583. 21440551 10.1016/j.jinsphys.2011.03.019

